# Analysis of radioprotection and antimutagenic effects of *Ilex paraguariensis* infusion and its component rutin

**DOI:** 10.1590/1414-431X20187404

**Published:** 2018-07-16

**Authors:** N. Bracesco, V. Sosa, L. Blanc, V. Contreras, E.C. Candreva, V.A. Salvo, S. Hocart, B. Mechoso, E. Nunes

**Affiliations:** 1 Radiobiology Laboratory, Department of Biophysics, Faculty of Medicine, University of the Republic, Montevideo, Uruguay; 2 Cancer Research Laboratory, Ponce School of Medicine Health Sciences, Ponce, Puerto Rico; 3 Cardiovascular Research Laboratory, Ponce School of Medicine Health Sciences, Ponce, Puerto Rico; 4 Peptide Research, Department of Medicine, Tulane University, New Orleans, LA, USA

**Keywords:** Radiation damage, *Ilex paraguariensis* infusion, Rutin, Radioprotection, Antimutagenic effects, Saccharomyces cerevisiae

## Abstract

DNA repair pathways, cell cycle checkpoints, and redox protection systems are essential factors for securing genomic stability. The aim of the present study was to analyze the effect of *Ilex paraguariensis* (Ip) infusion and one of its polyphenolic components rutin on cellular and molecular damage induced by ionizing radiation. Ip is a beverage drank by most inhabitants of Argentina, Paraguay, Southern Brazil, and Uruguay. The yeast *Saccharomyces cerevisiae* (*SC7Klys 2-3*) was used as the eukaryotic model. Exponentially growing cells were exposed to gamma rays (γ) in the presence or absence of Ip or rutin. The concentrations used simulated those found in the habitual infusion. Surviving fractions, mutation frequency, and DNA double-strand breaks (DSB) were determined after treatments. A significant increase in surviving fractions after gamma irradiation was observed following combined exposure to γ+R, or γ+Ip. Upon these concomitant treatments, mutation and DSB frequency decreased significantly. In the mutant strain deficient in *MEC1*, a significant increase in γ sensitivity and a low effect of rutin on γ-induced chromosomal fragmentation was observed. Results were interpreted in the framework of a model of interaction between radiation-induced free radicals, DNA repair pathways, and checkpoint controls, where the DNA damage that induced activation of MEC1 nodal point of the network could be modulated by Ip components including rutin. Furthermore, ionizing radiation-induced redox cascades can be interrupted by rutin potential and other protectors contained in Ip.

## Introduction

*Ilex paraguariensis* St. Hill., *Aquifoliaceae* (Ip) is a widely distributed tree in Southern Brazil, North-eastern Argentina, Paraguay, and Eastern Uruguay. Its dried leaves are used to prepare a traditional infusion (“mate”). The “mate” drinking habit has been popular for centuries, and was adopted from the native inhabitants of the region (“guaraní” Indians) (1).

In the past 25 years, the properties of Ip have been increasingly studied and the subject of several publications. Antioxidant properties (using chemical models and *ex vivo* lipoprotein studies), vase-dilating and lipid reduction properties, antimutagenic effects (depending on used model), anti-glycation effects, and weight reduction properties (2–9) have been reported.

Given the known effects of ionizing radiation as an inducer of reactive oxygen species (OH·, superoxide radicals, and H_2_O_2_) in intracellular media, we hypothesized that either Ip infusion or some of its components could counteract radiation damage at the cellular and molecular levels by trapping free radicals through modulation of DNA repair pathways. The putative protective effect could be detected *in vivo* by analyzing cell proliferation, mutagenesis, and DNA damage in irradiated cell populations of the eukaryotic model *Saccharomyces cerevisiae*.

Previous work at our laboratory showed that both caffeine and high temperatures had mutagenic effects on cell populations, which decreased significantly in the presence of Ip infusion (4 [Bibr B05] [Bibr B06] [Bibr B07] [Bibr B08]). This antimutagenic effect of Ip was attributed in part to the presence of B-complex vitamins in the infusion through modulation of error-free DNA repair (4,10,11). Furthermore, it was indicated that one or more Ilex components (i.e., polyphenols and vitamins) could induce cellular defense mechanisms such as DNA protection in a similar way as alpha-tocopherol (12,13).

The aim of present work was to analyze the putative protective role of Ip infusion (“mate”) and one of its polyphenolic components - the bioflavonoid rutin (quercetin-3-O-rutinoside) - on cellular and molecular damage induced by ionizing radiation. Importantly, the used concentrations simulated those found in the habitual infusion.

## Material and Methods

### Yeast strains and growth conditions

The following *Saccharomyces cerevisiae* haploid strains were used in the present analysis: *SC7K lys2-3*, wild type *SJR751* (*MATa ade2-101 his3∆200 ura3∆Nco lys2∆Bgl CAN1*), and the corresponding mutant strains: *sml1* (*MATa ade2-101 his3∆200 ura3∆Nco lys2∆Bgl CAN1 sml1∆::Kan*) and *sml1/mec1* (*MATa ade2-101 his3∆200 ura3 ∆Ncolys2∆Bgl CAN1 sml1∆::Kan mec1∆::Hyg*) (14).

Yeast cells were grown to exponential phase (N=1, 2×10^7^ cells per mL) at 30°C with aeration by shaking, in liquid nutrient medium YPD (1% yeast extract, 2% peptone, and 2% glucose (Sigma-Aldrich, USA).

### Extraction of “yerba mate” by liquid chromatography and mass spectrometry

Yerba mate (77.784 g, Canarias S.A., Uruguay) was added to hot water (70°C 500 mL) and mixed for 15 min. Filtrate was collected with a glass wool plug, and then filtered with a 0.2 µm filter for sterilization. The mate was concentrated by rotary evaporation under reduced pressure and lyophilized to yield a brown powder (22.17 g, 28.5%).

### Rutin analysis by liquid chromatography and mass spectrometry

Rutin hydrate (Sigma-Aldrich) was dissolved in methanol and diluted with water to give four separate standards containing 0.1, 0.125, 0.25 and 0.5 µg in 10 µL. Each standard (10 µL) was analyzed on a Shimadzu LCMS 2010A (Japan) using a diphenyl column (250 × 4.6 mm, 5 µm pore size, Vydac, (USA), a buffer system of 0.005% formic acid in water and B solution, and acetonitrile (0–50% B) over 30 min at a flow rate of 1 mL/min.

The dried Ip leaves (50 g) were added to 250 mL of distilled water at 70°C, and left to stand for 15 min, simulating the classic preparation. Thereafter, the infusion was sterilized with a fiberglass filter (2.7 µm). Final treatment concentration was 10%. Rutin hydrate (Sigma-Aldrich, USA) was diluted in distilled water, sterilized by filtration and used at the concentration found in the Ip infusion (40 µg/mL) (15). Both products at the indicated concentrations were added separately to liquid YPD cultures 1 h before irradiation (room temperature). Ip infusion was also added 5 min before irradiation in order to elucidate the kinetics of the putative protection. Exposure times corresponded approximately to 0.33 and 0.03 of cell cycle duration.

Irradiation was performed in Compton effect range, with a ^60^Co source (Nordion 220, (Canada), mean photon energy E=1.25 MeV, and dose rate of 13.4 kGy/h. The dosimetry was performed with a polymethyl methacrylate Harwell Amber S 3042 dosimeter (United Kingdom). Absorbed dose was 0 ≤D ≤200 Gy. Samples were irradiated in YPD liquid medium with or without Ip or R. After treatment, controls and treated samples were kept on ice before further processing.

**Figure 1.**
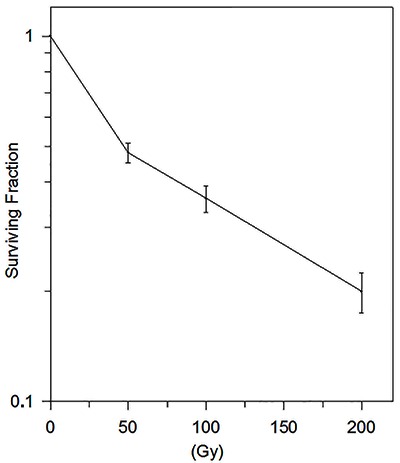
Surviving fractions as a function of γ-ray absorbed dose (Grays: Gy). Error bars indicate 95% binomial confidence intervals.

Relative surviving fraction was determined as a function of the absorbed doses. Based on survival curves (Figure 1) an absorbed dose of 200 Gy was selected for combination treatments. Aliquots of cells were plated in solid nutrient medium YPDA: YPD + 2% agar (DIFCO Laboratories, USA) and incubated at 30°C for 72 h. Survival was calculated as surviving fraction: S(x,y) = Ns/No, where Ns is the number of surviving cells capable of generating visible clones/mL; No is the total number of treated cells/mL; x the absorbed dose of radiation; and y the doses of the putative protectors (16,17).

To determine mutation frequency, cell samples of *SC7K lys2-3* were plated after treatment on omission media (OM: 2% dextrose, 0.67% nitrogenous base yeast (Sigma-Aldrich, USA), 2% agar) (17,18) and incubated at 30°C for 21 days. Thereafter, the number of revertants *lys* → *LYS* were scored (12,13). Mutation frequency M(x,y) and mutation yield Y(x,y) were calculated as: M(x,y) = Nm / Ns; Y(x,y) = Nm / No, where Nm is the number of mutants per mL, No is the number of treated cells per mL, x the absorbed dose, and y the dose of modulators (16,19,20).

### DNA double-strand breaks (DSB) determination

After exposure to treatments, nuclear DNA was isolated in agarose plugs after enzymatic treatment with lyticase and proteinase K (Sigma-Aldrich, USA) and submitted to pulse field electrophoresis (24 h, 3 steps; GeneLine II, Beckman, USA) (21 [Bibr B22]). Analysis of DNA bands was performed by ImageJ software (NIH, USA).

### Statistical analysis

Data from at least three reproducible experiments are reported as means±SD. Binomial or Poisson confidence limits were determined and plotted in figures. Where not shown, confidence limits are of the size of the symbols.

## Results

The analyzed samples showed high concentrations of polyphenols in Ip infusion as well as caffeine and other methylxanthines.

The characterization of Ip infusion and rutin quantification were performed using positive and negative ion mass chromatograms of Ip positive and negative ion; MS and UV spectra were recorded. Rutin eluted at 14.4 min and the area under the negative ion peak at 609.05 Da was determined to give a linear four-point calibration curve with an r^2^=0.9876 (Supplementary Figures S1, S2, and S3).

### Survival

**Figure 2.**
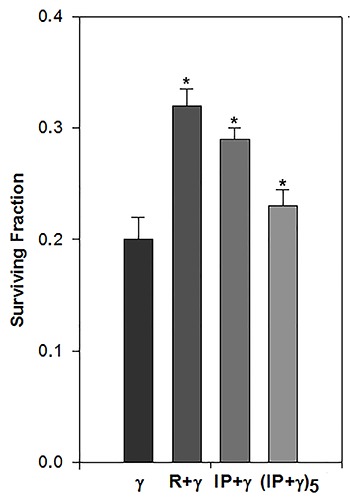
Surviving fractions of wildtype *SC7K lys2-3* cell samples exposed to gamma rays or to indicated combinations. γ: gamma ray of D=200 Gy; R+γ: rutin added 1 h before irradiation; Ip+γ: *Ilex paraguariensis* added 1 h before irradiation; (Ip+γ)_5_: *Ilex paraguariensis* added 5 min before irradiation. Data from at least three reproducible experiments are reported as means±SD. *P≤0.05 (binomial distribution).

Figure 1 shows surviving fraction as a function of γ-ray absorbed dose. An absorbed dose of 200 Gy giving a surviving fraction of 0.20±0.02 in *SC7K lys2-3* strain was selected for combined treatments of γ-rays either with Ip infusion or with rutin. The surviving fractions of irradiated (200 Gy) cells and respective controls are shown in Figure 2. A statistically significant increase was observed in cell survival after combined treatments compared to γ-ray acting as single agent. In the Ip+γ treatment group, the observed surviving fraction was significantly lower with addition 5 min before irradiation compared to addition 1 h before irradiation. The control samples and those treated either with Ip or with rutin showed similar surviving fractions (1.0±0.02).

**Figure 3.**
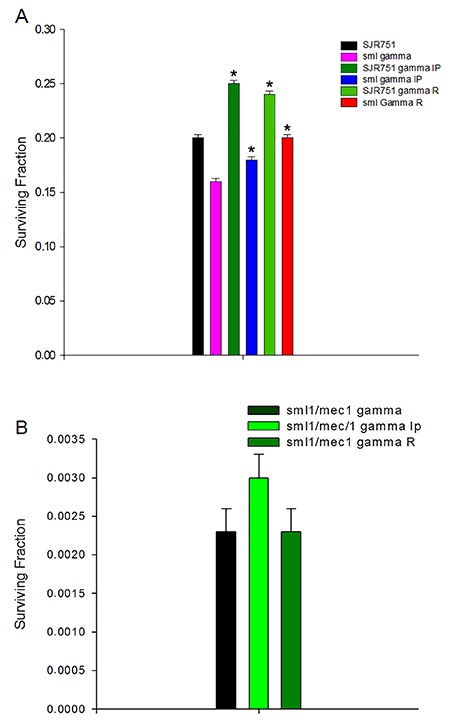
Surviving fractions of wild type *SJR751*, *sml1*, and *sml1/mec1* cell samples exposed to gamma rays (200 Gy), or to indicated combinations. Ip: *Ilex paraguariensis*; R: rutin. Data from at least three reproducible experiments are reported as means±SD. *P<0.05 (binomial distribution).

In the *SJR151* and *sml1* strains after 200 Gy exposure, surviving fractions were 0.20±0.02 and 0.16±0.03, respectively (Figure 3). Furthermore, both strains showed similar responses to γ treatment plus Ip and rutin as *SC7K lys2-3* (compare Figure 2 and 3). Meanwhile, and as expected, *sml1/mec1* mutant strain showed a significant increase in radiation sensitivity: S=0.0023±0.0028. The effects of γ exposure plus either Ip or rutin induced a low and not significant sensitivity increase compared to results using wild type *SC7K lys2-3* (Figure 3).

### Mutagenesis determination

**Figure 4.**
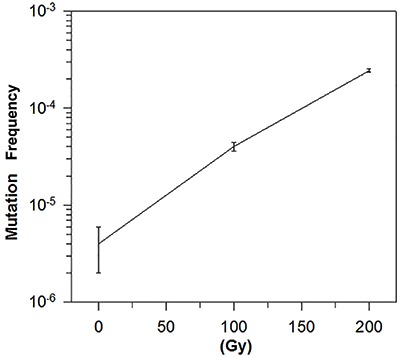
Mutation frequency of strain *SC7K lys2-3* as a function of absorbed gamma ray dose (Gy). Data from at least three reproducible experiments are reported as means±SD.

**Figure 5.**
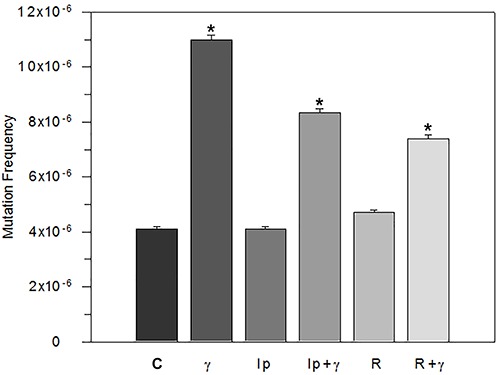
Mutation frequency of strain *SC7K lys2-3* after exposure to gamma rays (γ) at 200Gy and upon concomitant exposure to *Ilex paraguariensis* (Ip) and rutin (R). C: control group. Data from at least three reproducible experiments are reported as means±SD. *P<0.05 (binomial distribution).

In order to analyze the differential effect of each treatment on induced mutagenesis, cell samples of *SC7K lys2-3* were plated on omission medium, and mutation frequency as a function of absorbed dose was determined (Figure 4). An exponential increase in mutation frequency was observed. Figure 5 indicates the significant antimutagenic effect of rutin and Ip present in the nutrient medium one hour before irradiation (200 Gy).

### DNA double-strand breaks determination

**Figure 6.**
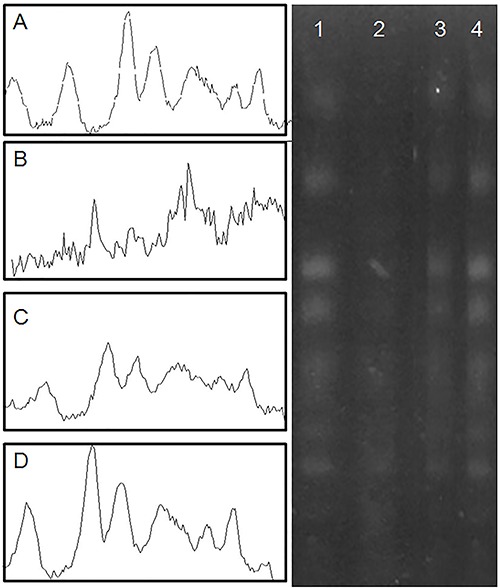
Densitograms (left) corresponding to samples shown in the right photograph from the *SC7K lys2-3* strain. Comparative absorbance (Y-axis) as a function of distance (X-axis). *A*: control sample; *B*: γ irradiated sample; *C*: γ + rutin treated sample, and *D*: γ + *Ilex paraguariensis* sample. Right panel: Chromosomal DNA gel photograph of treated and control cell samples after pulsed field electrophoresis. From left to right: 1: control sample; 2: γ irradiated sample; 3: γ + rutin treated sample; 4: γ + *Ilex paraguariensis* sample.

Cell samples irradiated in the aforementioned conditions and respective controls were submitted to pulsed field electrophoresis after DNA isolation. In *SC7K lys2-3*, Ip as well as rutin resulted in protection against DNA breakage if present during irradiation. Thus, a decrease in γ-ray induced DSBs was observed, associated with the increased surviving fractions. Importantly, neither Ip nor rutin induced significant DSBs compared to the untreated control sample (Figure 6).

To elucidate the role of Ip and rutin in the observed increase in radiation resistance, we analyzed the corresponding effect on chromosomal fragmentation after irradiation in the mutant strain *sml1/mec1* defective in *MEC1/hATR* gene.

**Figure 7.**
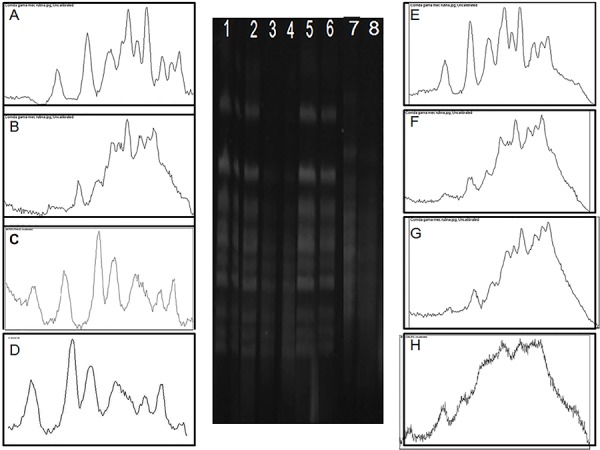
Densitograms corresponding to the *SJR751* strain (*A*–*D*) and *sml1/mec1* strain (*E*–*H*) shown in the 1–8 middle panel photograph, respectively. Comparative absorbance (Y-axis) as a function of distance (X-axis). *A and E*: control sample; *B and F*: γ irradiated sample; *C and G*: γ + rutin; *D and H*: γ + *Ilex paraguariensis* sample. Middle panel: Chromosomal DNA gel photograph of control and treated cell samples after pulsed field electrophoresis.

Regarding induced DSB after 200Gy, no significant effect of either rutin or Ip was observed compared to the corresponding wild type as well as to the *sml* strain (Figure 7).

## Discussion

The present results showed that Ip infusion and Ip component rutin had a significant protective effect against ionizing radiation in cell populations of haploid *S. cerevisiae* if added 1-h before irradiation and during it at concentrations corresponding to the habitual mate infusion. Importantly, Ip infusion showed relatively high concentrations of polyphenols including rutin. In fact, both natural products induced a significant increase in the survival probability of irradiated cell populations, as well as a significant decrease in the mutation frequency and in DSBs induced by gamma irradiation. Shorter exposure times to the infusion or to rutin showed a lower effect. Importantly, the spontaneous mutation frequency was not significantly altered upon single treatments.

Ionizing radiation at used energies and in aqueous nutrient media induced primary and secondary ionizations determining the formation of free radicals (reactive oxygen species and reactive nitrogen species - ROS and NRS) and oxidative damage in proteins, lipids, and DNA. These types of damage have been extensively investigated in different contexts (22 for review). Part of these modifications are reversible through red-ox mechanisms, or can be repaired by enzymatic networks, and part undergo cell death (lack of proliferation ability in optimal conditions, Poisson distribution). Induced lethal genomic events (including DSB, chromosomal aberrations, and lethal mutations) in eukaryotic cells depend on a linear quadratic relationship in absorbed dose, where the probability of repair is an interactive factor (23).

In case of mutant strain *sml1/mec1* deficient in *MEC1*, a significant increase in radiation sensitivity was observed. Regarding surviving fractions and chromosomal fragmentation after gamma exposure upon Ip or rutin addition, a low, not significant effect was observed, indicating the important role of *MEC1* in the observed radiation response.

Other authors have shown that resveratrol, a polyphenol synthesized in several plants including *Ilex paraguariensis*, can modulate DSB repair and proliferation in lymphoblastic cell lines. It was suggested that this modulation depends on *ATM/ATR-p53* cell cycle control and repair pathways (24 [Bibr B25]). Importantly, these pathways have structural and functional homology between animal cells and yeast. However, other authors found an increase in chromosomal aberrations and the induction of micronuclei after resveratrol exposure (24–26). The different and somewhat contradictory effects of some plant components on cell death and genomic stability could depend on the use of different media and concentrations (27 [Bibr B28] [Bibr B29]). Several studies have indicated an important role of natural products, such as tea and its polyphenolic components, as radical scavengers, antioxidants, and anti-inflammatory chemopreventive agents. Tea infusions (*Camellia sinensis*) and tea polyphenols have been shown to inhibit tumor growth at different localizations and in several animal models. This inhibition was associated with decreased cell proliferation, increased apoptosis, and modulation of transduction cascades (27–30 for review). Thus, the potential use of tea, polyphenols, and other tea-derived products for cancer prevention is presently in continuous investigation. In this context, it was reported that peroxynitrite and lipoxygenase-induced oxidation in human LDL is inhibited by “mate” extracts and quercetin (2,31–33).

The present results indicated the central role of the phosphoinositol *(PI3)* kinase *Mec1* in the activation of repair pathways involved in radiation resistance and their increase in the presence of Ip and rutin observed in wild type. Presence of these natural products previously to and during irradiation is essential to explain the observed increase in radiation resistance that depends on DNA repair transfunctions and protection through radical scavenging.

Alternative checkpoints function (*CHK1* and *RAD53*) determine division delay and activate DSBs repair pathways (homologous recombination and non-homologous end-joining) (14,34,35 [Bibr B36]). Our results indicated the important role of *PI3* kinase *Mec1* in genome stability after radiation damage. In fact, upon mutation of *MEC1*, an increase in radiosensitivity, as measured by surviving fraction, and a significant increase in DSB induction was observed. Furthermore, and regarding radiation resistance, error-prone or error-free *Rad6* and *Rad18* dependent trans-lesion synthesis (TLS) play an important role upon single and double strand breaks as well as upon base damage (21,24,30). Induced base oxidative damage (i.e., 8-oxoG) in DNA is counteracted by base excision repair (BER), as well as by components of mismatch repair (MMR) and nucleotide excision repair (NER) pathways in connection to the recombination repair pathway (14,30 for review, 34–37).

The antioxidants contained in the Ip infusion can partially change the generation and fate of free radicals induced by radiation, depending on their concentration and redox potential of the involved components. The interference at redox cascades can take place at different compartments (i.e., at mitochondrial, and nuclear levels) as well as in the extracellular medium. The present results showed that the addition of Ip 5 min before irradiation only provided a mild protective effect, indicating that the time required for transport and intracellular distribution of the antioxidants play an important role in the observed radioprotective effects.

Based on the present and previous results on radiation resistance pathways, it is proposed that *PI3* kinase *Mec1* acted as a nodal point in the regulatory network of transduction events elicited by direct and indirect oxidative effects of ionizing radiation. Importantly, it is known that *ATR* is a human *MEC1* homolog.

Since protection against ionizing radiations and DNA repair are current important topics regarding human health and radioecology, more investigations are needed to further elucidate the involved mechanisms at molecular, cellular, and system levels.

## Supplementary material

Click here to view [pdf].
